# Predictive Biomarker for Cardiac Surgery-Associated Acute Kidney Injury: A Retrospective Analysis

**DOI:** 10.21470/1678-9741-2024-0192

**Published:** 2025-09-11

**Authors:** Bengui Zhang, Dayong Zhang, Shidong Wang, Hongbo Yu

**Affiliations:** 1 Department of Cardiovascular Surgery, Guangyuan Central Hospital, Guangyuan City, Sichuan Province, People’s Republic of China

**Keywords:** Acute Kidney Injury, Biomarkers, Lipocalin-2, Interleukin-2, Tumor Necrosis Factors, Cardiac Surgical Procedures.

## Abstract

**Introduction:**

Cardiac surgery-associated acute kidney injury (CSA-AKI) is a popular and severe complication after cardiac surgery. We aimed to set up a quick and accurate predictive model for rapid identification of CSA-AKI and to evaluate its predictive value.

**Methods:**

In this retrospective study, we included a total of 120 patients who underwent heart surgery and divided them into 55 patients who developed kidney injury following heart surgery (CSA-AKI group) and 65 patients who did not experience kidney injury after the same surgical procedure (non-CSA-AKI group). The predictive capacity of various laboratory indicators for CSA-AKI were assessed, including tumor necrosis factor-α (TNF-α), interleukin 2, interleukin 6, and neutrophil gelatinase-associated lipocalin (NGAL). Additionally, receiver operating characteristic curve analysis was employed to evaluate the performance of the model in predicting CSA-AKI.

**Results:**

After cardiac surgery, patients who developed CSA-AKI exhibited significantly higher levels of TNF-α, interleukin 2, interleukin 6, and NGAL compared to the control group. Receiver operating characteristic curve analysis revealed that TNF-α, interleukin 2, interleukin 6, and NGAL showed good diagnostic performance, with area under the curve values of 0.66, 0.78, 0.66, and 0.80, respectively. Further analysis demonstrated that the combination of TNF-α, interleukin 2, interleukin 6, and NGAL had the highest predictive value for acute kidney injury (area under the curve = 0.93).

**Conclusion:**

TNF-α, interleukin 2, interleukin 6, and NGAL exhibited a promising predictive capability for CSA-AKI, while a combined diagnostic model was established to enhance the diagnostic value further.

## INTRODUCTION

**Table:** 

Abbreviations, Acronyms & Symbols	
ACEI/ARB	= Angiotensin-converting enzyme inhibitor/angiotensin receptor blocker
AKI	= Acute kidney injury
AUC	= Area under the curve
CABG	= Coronary artery bypass grafting
CI	= Confidence interval
CPB	= Cardiopulmonary bypass
CSA-AKI	= Cardiac surgery-associated acute kidney injury
ECMO	= Extracorporeal membrane oxygenation
eGFR	= Estimated glomerular filtration rate
IABP	= Intra-aortic balloon pump
IL	= Interleukin
KDIGO	= Kidney Disease: Improving Global Outcomes
NGAL	= Neutrophil gelatinase-associated lipocalin
NK	= Natural killer
ROC	= Receiver operating characteristic
TNF-α	= Tumor necrosis factor-α

Acute kidney injury (AKI) is a usual and significant complication after cardiac surgery^[1]^. In recent years, according to the clinical definition of different diagnostic criteria for AKI, the incidence of cardiac surgery-associated acute kidney injury (CSA-AKI) has ranged between 20% and 40%, and 1.6% to 5.8% of patients after cardiac surgery require renal replacement therapy^[2]^. According to the Kidney Disease: Improving Global Outcomes (KDIGO) standard classification, the incidence of KDIGO standard grades 1, 2, and 3 was 13.6%, 3.8%, and 2.7%, respectively^[3]^. The prevalence of CSA-AKI diagnosed via Acute Kidney Injury Network and KDIGO criteria was 28% and 24.2%, respectively, significantly higher than that diagnosed by the Risk, Injury, Failure, Loss, End-stage kidney disease (or RIFLE) criteria (18.9%)^[4]^. AKI occurs most often during or after cardiac surgery and is most common within two days after surgery^[5]^. Aorta occlusion, blood ultrafiltration, centrifugal pump use, and pulsating perfusion during cardiopulmonary bypass (CPB) in traditional cardiac surgery can promote inflammation, cause renal small vessel contraction and microthrombus formation, and induce postoperative AKI. The short-term mortality rate of CSA-AKI is between 16% and 31%, while the mortality rate of CSA-AKI with renal replacement therapy can be as high as 50-80%^[6]^. Studies have shown that mildly elevated serum creatinine during hospitalization is an independent risk factor for long-term end-stage renal disease and death. In addition, several studies have shown that AKI, even when kidney function is fully restored, increases the risk of long-term death^[7-9]^. In a prospective follow-up study, two-year mortality and the incidence of progressive chronic kidney disease increased significantly after renal function had fully recovered in heart surgery patients^[10]^. Therefore, it is of great significance to establish a diagnostic model for early detection and rapid identification of CSA-AKI to help clinicians quickly identify and treat patients with CSA-AKI. Therefore, rapid identification of the occurrence of CSA-AKI is of great significance for early and timely postoperative monitoring, subsequent diagnosis, and treatment.

A slight increase in creatinine in the early postoperative period can seriously affect the prognosis of patients. Early diagnosis of CSA-AKI is needed because serum creatinine changes are not dynamic enough^[11]^. Therefore, new strategies for kidney protection are needed in patients undergoing heart surgery. Studies have shown that AKI-related biomarkers such as kidney injury molecule-1^[12,13]^, neutrophil gelatinase-associated lipocalin (NGAL)^[14]^, and interleukin (IL) 6^[15]^ are direct and more specific indicators of kidney injury^[16]^. Elevated NGAL levels are often a good predictor of the need for kidney replacement after severe kidney injury^[17]^. In addition, NGAL is known to predict mortality from kidney disease, and elevated levels of NGAL are often effective predictors of the need for kidney replacement after severe kidney injury^[18,19]^. Pro-inflammatory cytokines play a crucial character in these mechanisms of kidney injury. The proliferation of the immune response occurs when white blood cells in the blood come into contact with the artificial surface of the extracorporeal circulation system under the action of IL-6, IL-2, and tumor necrosis factor-α (TNF-α)^[20,21]^. The enhanced immune response and increased oxidative stress (secondary to *in vitro* oxygenation) exacerbate the disruption of microcirculation in the renal tubule arterioles, leading to ischemia within these structures^[22]^. Therefore, perioperative monitoring of cardiac and renal function indexes is very important to accurately predict postoperative renal injury and take corresponding treatment measures.

In this investigation, we examined pertinent laboratory biomarkers linked to CSA-AKI, namely TNF-α, IL-2, IL-6, and NGAL, assessing their levels preand post-cardiac surgery. Leveraging these biomarkers, we developed a comprehensive diagnostic model to promptly identify CSA-AKI. The diagnostic model crafted in this research offers healthcare practitioners swift and precise perioperative patient status insight, facilitating informed clinical decision-making.

## METHODS

### Study Design and Participants

This research work is a single-center retrospective study. Adult patients undergoing heart surgery in our hospital were enrolled from 2016 to 2022. This research work has been approved by the Ethics Committee of our Institute (2025-018) and is following the Declaration of Helsinki^[23]^. Informed consent was waived for this retrospective study due to the exclusive use of de-identified patient data, which posed no potential harm or impact on patient care.

Inclusion criteria: (1) adult (aged 18 years or older); (2) patients undergoing heart surgery in our hospital from May 2016 to October 2022; (3) the clinical information of patients was relatively complete.

Exclusion criteria: (1) subjects with baseline renal dysfunction (estimated glomerular filtration rate [eGFR] < 60 ml/min*1.73m2); (2) kidney replacement therapy before cardiac surgery; (3) history of unilateral nephrectomy; (4) significant fluctuations in serum creatinine levels within seven days before surgery (defined as a change of ≥ 0.3 mg/dL or ≥ 50% from baseline), to exclude patients with other acute or chronic renal impairment processes that could confound CSA-AKI assessment; (5) ventricular tachycardia or ventricular fibrillation before surgery; (6) cardiac arrest before surgery requiring cardiopulmonary resuscitation; (7) tracheal intubation mechanically-assisted ventilation performed before surgery and remained on the operating room, (8) extracorporeal membrane oxygenation (ECMO) or intra-aortic balloon pump (IABP) used before surgery and continued until the operating room.

### Settings

The patients were divided into two groups: the CSA-AKI group, consisting of 55 patients who developed kidney injury following heart surgery, and the non-CSA-AKI group, consisting of 65 patients who did not experience kidney injury after the same surgical procedure. The primary clinical endpoint of this study was the occurrence of AKI following cardiac surgery, with a focus on both mild AKI and moderate to severe AKI as the two concurrent primary clinical endpoints. The diagnostic criteria for contrast-induced AKI were based on the 2012 KDIGO criteria^[24]^, which stipulate that post-cardiac surgery, patients exhibit a serum creatinine increase of no < 0.3 mg/dl within 48 hours or > 1.5 times the rise in serum creatinine from the preoperative baseline within seven days after cardiac surgery. We chose the KDIGO criteria due to its high sensitivity and widespread clinical use, providing a reliable basis for the early detection and intervention of AKI.

### Data Collection

The clinical information of enrolled patients were extracted from the electronic medical record system of our hospital. Demographic characteristics (age, sex) and comorbidities (hypertension, diabetes, baseline creatinine and laboratory indicators: IL-2, IL-4, IL-6, IL-10, TNF-α, IFN-r, IL-17A, NGAL, etc.) were obtained. The laboratory tests were the results of cardiac surgery after admission. If there were multiple times, the last results before surgery were included for analysis. Information on medication used by patients before surgery was retrieved. The baseline eGFR was calculated using the Chronic Kidney Disease-Epidemiology Collaboration formula based on baseline serum creatinine values. The baseline serum creatinine value was the lowest at three months before hospitalization. If the creatinine value was not available before admission, the lowest serum creatinine value after admission and before surgery should be used.

In this study, we collected information on the types of cardiac surgeries performed on all included patients. The types of surgeries included coronary artery bypass grafting (CABG), valve replacement or repair, aortic surgeries (*e.g.*, aneurysm repair), and other complex cardiac surgeries. We also recorded whether supportive techniques such as CPB, ECMO, and IABP were used during each procedure. This information aims to provide an overview of the different types of surgeries and their complexity in relation to the incidence of CSA-AKI.

### Statistical Analyses

IBM Corp. Released 2019, IBM SPSS Statistics for Windows, version 26.0, Armonk, NY: IBM Corp. software was employed for the analyses. Measurement data following a normal distribution were presented as mean ± standard deviation, while categorical data were presented as frequencies or percentages. Continuous variables were compared using *t*-tests or Wilcoxon rank-sum tests, and categorical data were compared using the chi-square test or Fisher's exact method. Due to a high level of correlation coefficients among various CSA-AKI biomarkers, we utilized principal component analysis to combine the postoperative biomarkers. The principle behind principal component analysis involves orthogonal rotation, converting highly correlated variables into uncorrelated principal components. We considered retaining components with an eigenvalue of a matrix > 1 and a variance explained > 10%. Additionally, we conducted a screen test to determine which components to retain.

## RESULTS

### The Baseline of Clinical Characteristics

The baseline characteristics of 120 patients revealed no statistically significant differences between the CSA-AKI group and non-CSC-AKI group, as delineated in Table 1. There existed no substantial variances between the two cohorts concerning average age (59.65 ± 3.29 years *vs.* 56.35 ± 1.79 years) or sex distribution (45.45% female *vs.* 49.23% female). Furthermore, an in-depth analysis of the essential medical histories encompassing hypertension, diabetes, preoperative creatinine values, and medication regimens was conducted across both study populations. The findings exhibited negligible disparities in the fundamental medical profiles (*P* > 0.05). The congruity in baseline characteristics implies comparability between the selected cohorts, thereby mitigating potential confounders that could impact the study's outcomes. Additionally, Table 2 presents the impact of different types of cardiac surgeries on the incidence of CSA-AKI. The data shows that aortic surgeries have the highest incidence of CSA-AKI, reaching 62.5%, while the incidence for CABG is lower at 20.68%. The incidence rates for valve surgeries and other complex cardiac surgeries are 40.74% and 47.36%, respectively. These data illustrate the diversity of surgery types and the occurrence of CSA-AKI.

**Table 1:** Baseline characteristics of patients.

Characteristic	CSC-AKI group (n = 55)	Non-CSC-AKI group (n = 65)	*P*-value
Age (mean ± standard deviation)	59.65 ± 3.294	56.35 ± 1.789	0.9445
Female, n (%)	25 (45.45%)	32 (49.23%)	0.9999
Male, n (%)	30 (54.55%)	33 (50.77%)	0.9999
History, n (%)			
Hypertension	23 (41.82%)	37 (56.92%)	0.6667
Diabetes	14 (25.45%)	10 (15.38%)	0.6858
Preoperative creatinine values (umol/L)	118.4 ± 15.16	102.60 ± 36.01	0.886
ACEI/ARB (48 h before surgery)	29 (52.28%)	30 (46.15%)	0.999
Statins (24 h before surgery)	30 (54.55%)	35 (53.85%)	0.999

Data are presented as mean ± standard deviation or n (%)

ACEI/ARB=angiotensin-converting enzyme inhibitor/angiotensin receptor blocker; CSA-AKI=cardiac surgery-associated acute kidney injury

**Table 2:** Incidence of CSA-AKI based on cardiac surgery types.

Type of surgery	Patients	CSA-AKI cases	CSA-AKI (%)
Coronary artery bypass grafting	58	12	20.68%
Valve replacement or repair	27	11	40.74%
Aortic surgery (*e.g.*, aneurysm repair)	16	10	62.5%
Other complex cardiac surgeries	19	9	47.36%

CSA-AKI=cardiac surgery-associated acute kidney injury

### Comparison of the Levels of IL-2, IL-6, TNF-α, and NGAL in Patients with Acute Kidney Injury Before and After Cardiac Surgery

**Fig 1:**
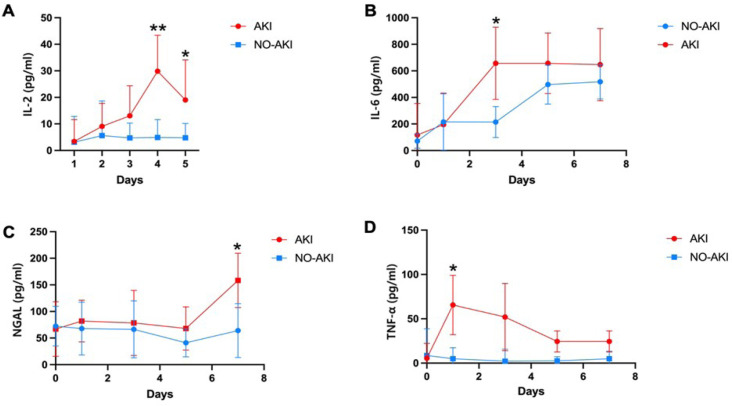
Alteration of indicators between the cardiac surgery-associated acute kidney injury (CSC-AKI) group and the non-CSC-AKI group after cardiac surgery. (A) Interleukin (IL)-2, (B) IL-6, (C) tumor necrosis factor-α (TNF-α), (D) neutrophil gelatinase-associated lipocalin (NGAL). *P < 0.05, **P < 0.01, CSC-AKI group vs. non-CSC-AKI group.

Through analysis of variance, we identified AKI-related parameters for detailed investigation, as shown in Figure 1. Following surgery, the results demonstrated significant elevations in levels of IL-2, IL-6, TNF-α, and NGAL for the CSC-AKI group, whereas only the levels of IL-6 exhibited a statistically significant increase, with the other indicators showing minimal fluctuation but no significant change for non-CSC-AKI group. Notably, for the CSC-AKI group, IL-2 peaked on the fifth postoperative day before gradually declining by the seventh day. IL-6 demonstrated consistent elevation in both AKI and control cohorts; however, the rise in IL-6 levels was more pronounced in AKI patients compared to non-AKI individuals. In the CSC-AKI cohort, TNF-α surged rapidly at the onset of surgery, tapering off by the seventh day. Moreover, NGAL levels in AKI patients began rising on the seventh day, contrasting with no observable changes in the control group. This detailed examination underscores the dynamic nature of these biomarkers in the context of AKI and provides valuable insights into their temporal patterns and potential clinical significance.

### The Diagnosis Ability of Biomarkers for Cardiac Surgery-Associated Acute Kidney Injury Patients

**Fig 2:**
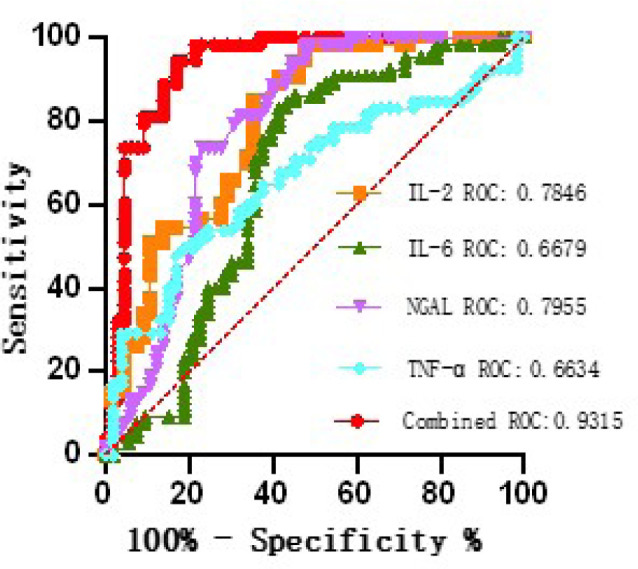
Receiver operating characteristic (ROC) curve of acute kidney injury-related indicators. IL=interleukin; NGAL=neutrophil gelatinase-associated lipocalin; TNF-α=tumor necrosis factor-α.

We preliminarily analyzed the diagnostic efficacy of TNF-α, IL-2, IL-6, and NGAL for CSA-AKI respectively, but the results were feebly satisfactory, as illustrated in Table 3 and Figure 2. Therefore, the maximum point of Youden index (sensitivity + specificity - 1) is taken as the best cutoff value, and the maximum sensitivity and specificity of biomarkers (TNF-α, IL-2, IL-6, NGAL) in CSA-AKI detection is calculated by the abscissa and ordinate of corresponding receiver operating characteristic (ROC) curve. The diagnostic value of TNF-α, IL-2, IL-6, and NGAL in patients with CSA-AKI was evaluated by the size of the area under the curve (AUC = 0.9315, 95% confidence interval [CI] = 0.8831 to 0.9799) of the ROC curve, the ideal cutoff value, and the corresponding sensitivity and specificity.

**Table 3:** Receiver operating characteristic diagnostic performance of each index

Variable	Sensitivity, %	Specificity, %	Cutoff value	AUC	95% CI	*P*-value
TNF-α	60	65	0.25	0.66	0.57 to 0.76	0.004
IL-2	70.51	60.26	0.3	0.78	0.70 to 0.87	< 0.001
IL-6	66.67	60.26	0.27	0.66	0.56 to 0.77	< 0.001
NGAL	65	96.25	0.61	0.80	0.72 to 0.87	< 0.001
Combined diagnosis	85	95	0.8	0.93	0.88 to 0.98	< 0.001

AUC=area under the curve; CI=confidence interval; IL=interleukin; NGAL=neutrophil gelatinase-associated lipocalin; TNF-α=tumor necrosis factor-α

## DISCUSSION

Up to the present time, there are still no uniform criteria for the quick and accurate prediction of CSA-AKI occurrence, although the modified definition of AKI recognized via the KDIGO crowd is extensively employed^[25]^. In this research study, we found an unusual preoperative extrapolative model that recognized patients with a high risk of CSA-AKI using commonly measured clinical parameters obtained before surgery (AUC = 0.9315, 95% CI: 0.8831 to 0.9799) for CSA-AKI. In brief, this potential diagnosis model can postulate clinical assistance for early recognition, diagnosis, and even intervention for CSA-AKI.

Unfortunately, the pathogenic and physiological mechanism of CSA-AKI is the result of numerous corridors of interaction and cannot be explained via a definite pathogeny. In this work, we investigated that preoperative serum IL-6 and IL-2 levels were associated with AKI development. Patients with CSA-AKI always have a higher secrete level of IL-6 and IL-2 in seven days after surgery than those without AKI. IL-6 has been found to be created directly in the injured tissue during the early stages of the inflammatory process^[26]^. What’s more, IL-6 stimulates differentiation and triggers T cells, B cells, macrophages, and other immune-associated cells in response to the damaged tissue^[27]^. The pleiotropic effects of IL-6 in the inflammatory-related immune effect include stimulating the production of acute phase proteins (C-reactive protein, fibrinogen), as well as inhibiting the synthesis of albumin, fibronectin, and transferrin^[28]^. Additional proof as a bridge linking IL-6 and kidney disease is that eGFR is inversely associated with circulating stages of pro-inflammatory biomarkers^[29]^. Subjects with higher levels of inflammatory biomarkers than the control groups had significantly lower eGFR^[30]^. Serum IL-6 levels of patients before surgery showed a significant correlation with the prognosis of postoperative AKI. What’s more, the subjects with IL-6 concentrations in the upper quartile had a six-fold greater risk of developing stage II and III AKI than patients with the secretion of IL-6^[31]^. The abovementioned evidence focuses on the important character of inflammation during the pathophysiology change of AKI. Interestingly, we discovered that patients who have higher preoperative levels of serum IL-6 were individually associated with a greater likelihood of the development of AKI. In conclusion, the present evidence revealed that serum IL-6 tested preoperatively, as a crucial forecast biomarker of inflammation, can serve as clinically relevant guidance of the monitor factor of AKI.

The initial onset of AKI exhibited an exact correlation with extraordinary levels of peripheral interleukins (IL-2, IL-6, IL-8, IL-18) in plasma^[26]^. Continued secretion and accumulation of the inflammatory factors can cause severe damage to the kidney^[28]^. Therefore, the removal of inflammatory factors timely is an important measure of a positive protective effect on renal function. IL-2 is chiefly synthesized via CD4+ T cells afterwards antigen or mitogen stimulation and is also a cytokine with pleiotropic effects^[32]^, which take part in the immune response and protection against viral infection. Meanwhile, IL-2 also take part in triggering T cells to promote cytokine secretion and stimulate natural killer (NK) cell proliferation enhancing the cytotoxic vigour and assembly of cytokines via NK cells. This physiological process involves the generation of lymphokine-activated killer cells, which also supports B cell proliferation and Ab secretion and sensitizes macrophages^[33]^. In addition, NGAL has been acknowledged as a primary biomarker of AKI. The circulating NGAL in plasma has been elevated because of decreased glomerular filtration, whereas urinary NGAL meaningfully amplified because of declined proximal tubule reabsorption and upregulation of NGAL expression from the loop of Henle and the distal tubule^[34,35]^. These inflammation-related biomarkers have been researched extensively in the early discovery and forecast of the progression of AKI in various settings^[28,36]^. Under the operation of inflammatory-related cytokines, the cells in the renal tubular manufacture MCP-1 to attract monocytes and tissue macrophages, which also mediate the changes in the expression of TNF-α^[37]^. Our consequence in this work post-cardiac surgery described that a higher concentration of TNF-α in plasma was obviously connected with an essential risk factor of incident CSA-AKI.

### Limitations

It is important to acknowledge several limitations of this study. Firstly, it is a single-center retrospective study, which may limit the generalizability of the findings to other populations. Validation in diverse populations is necessary to accurately assess the stability of the identified risk factors. Secondly, despite utilizing strict statistical methods and relying on biochemical markers, there is still a possibility of bias in the results. The model developed in this study was based on a small sample size, highlighting the need for further multi-center studies to verify the predictive efficacy of the diagnostic model. Additionally, the enrolled cases involved complex surgical procedures, introducing numerous influencing factors during the perioperative period. These factors should be considered when interpreting the results and may impact the generalizability of the findings.

## CONCLUSION

In summary, the predictive model established in this study showed good predictive efficacy in identifying the occurrence of postoperative AKI. The indicators included in the model were all those that could be quickly obtained from laboratory tests, which is very important for real-time detection of intraoperative and postoperative status of cardiac patients. In addition, our predictive model has important guiding implications for helping clinicians make rapid treatment decisions for AKI in patients with heart disease.
